# Long-acting injectable antipsychotics vs schizophrenia: a descriptive study in a Greek university hospital

**DOI:** 10.1192/j.eurpsy.2025.2097

**Published:** 2025-08-26

**Authors:** G. N. Porfyri, E. Kottaridou, V. Tarantili, A. Konsta

**Affiliations:** 1 National and Kapodistrian University of Athens, School of Medicine, MSc “Global Health-Disaster Medicine”, Athens; 2 1st Psychiatric Clinic, General Hospital of Papageorgiou, Thessaloniki; 3 General Hospital of Argos, Argos, Greece

## Abstract

**Introduction:**

Antipsychotics’ adverse effects in combination with patients’ anosognosia, which is frequent among individuals with schizophrenia, lead to high rates of medication nonadherence. However, long-acting injectable (LAI) antipsychotics represent a veritable ally to the eyes of patients suffering from schizophrenia. Instead of the daily pill-taking required with oral antipsychotics, LAI antipsychotics are administered by injection at two- to four-week intervals, permitting patients to feel more independent, self-secure and free.

**Objectives:**

To explore the sociodemographic profile of patients receiving LAIs and to highlight the positive and the negative impact this treatment had on their health.

**Methods:**

The study sample consisted of 44 patients followed-up in the Depot Outpatient Department of Papageorgiou General Hospital in Greece. The research was conducted between 2023 and March 2024. The sample was divided into subgroups according to gender, diagnoses - according to the International Classification of Diseases (ICD-10)-, and type of long-acting antipsychotic treatment. A bivariate analysis was performed to examine relationships between variables, such as: (a.) age; (b.) family status (c.) BMI; (d.) number of lifetime hospitalizations; (e.) lifetime suicide attempts.

**Results:**

63.6% of patients were men, 36.4% were females.

90.9% were diagnosed with Schizophrenia (F20).

31% were between 31 - 40 years old, while 26.2% were between 51 - 60 years old.

61.4% were unmarried, while 13.6% were married and 13.6% were divorced.

81.8% were unemployed/receiving welfare benefits.

68.2% lived with a relative.

56.8% claimed not suffering from physical diseases. However, when physical disorders were reported, they mainly included dyslipidaemia, diabetes and hypertension.

Based on their BMI, 37.2% were in the 2nd degree of obesity, 25.6% were in the 1st degree of obesity and 30.2% had normal weight.

47.7% were on olanzapine, 22.7% were on paliperidone and 11.4% were on haloperidol or aripiprazole.

The average value of years on LAI treatment was 3.5 years, with a minimum of 1 month and a maximum of 12 years.

Prior LAI treatment, the average value of hospitalizations was 3.5, with a minimum of 1 hospitalization and a maximum of 21 hospitalizations. After receiving treatment, 95.5% of patients were never hospitalized.

Prior LAI treatment, 88.6% of patients had no history of suicide attempts, while 11.4% had one or two suicide attempts. After receiving treatment, no participant had any suicide attempt.

**Conclusions:**

Long-acting injectable antipsychotics help patients to live their lives outside of a psychiatric ward, by drastically diminishing the number of hospitalizations as well as the number of suicide attempts. But when it comes to their physical health, patients face many adverse effects, such as obesity. Clinicians must stay vigilant to ensure the quality of physical health of their patients.

**Disclosure of Interest:**

None Declared

